# Automated Decision Support For Community Mental Health Services Using National Electronic Health Records: Qualitative Implementation Case Study

**DOI:** 10.2196/35403

**Published:** 2022-07-05

**Authors:** Yasmin van Kasteren, Jörg Strobel, Tarun Bastiampillai, Ecushla Linedale, Niranjan Bidargaddi

**Affiliations:** 1 Flinders Health and Medical Research Institute College of Medicine and Public Health Flinders University Adelaide Australia; 2 Flinders Digital Health Research Centre Flinders University Adelaide Australia; 3 Barossa Fleurieu Adelaide Hills Local Health Network South Australia Australia; 4 Health Translation SA South Australian Health and Medical Research Institute Adelaide Australia

**Keywords:** implementation, computerised clinical decision system, decision system, decision support, participatory action framework, psychotropic medication, psychotropic, nonadherence, monitoring, medication adherence, algorithms, algorithm, electronic health records, EHR, health record, normalization process theory, automated alerts, automated alert, mental health, mental illness, adherence, medication, eHealth, web-based

## Abstract

**Background:**

A high proportion of patients with severe mental illness relapse due to nonadherence to psychotropic medication. In this paper, we use the normalization process theory (NPT) to describe the implementation of a web-based clinical decision support system (CDSS) for Community Mental Health Services (CMHS) called Actionable Intime Insights or AI^2^. AI^2^ has two distinct functions: (1) it provides an overview of medication and treatment history to assist in reviewing patient adherence and (2) gives alerts indicating nonadherence to support early intervention.

**Objective:**

Our objective is to evaluate the pilot implementation of the AI^2^ application to better understand the challenges of implementing a web-based CDSS to support medication adherence and early intervention in CMHS.

**Methods:**

The NPT and participatory action framework were used to both explore and support implementation. Qualitative data were collected over the course of the 14-month implementation, in which researchers were active participants. Data were analyzed and coded using the NPT framework. Qualitative data included discussions, meetings, and work products, including emails and documents.

**Results:**

This study explores the barriers and enablers of implementing a CDSS to support early intervention within CMHS using Medicare data from Australia’s national electronic record system, My Health Record (MyHR). The implementation was a series of ongoing negotiations, which resulted in a staged implementation with compromises on both sides. Clinicians were initially hesitant about using a CDSS based on MyHR data and expressed concerns about the changes to their work practice required to support early intervention. Substantial workarounds were required to move the implementation forward. This pilot implementation allowed us to better understand the challenges of implementation and the resources and support required to implement and sustain a model of care based on automated alerts to support early intervention.

**Conclusions:**

The use of decision support based on electronic health records is growing, and while implementation is challenging, the potential benefits of early intervention to prevent relapse and hospitalization and ensure increased efficiency of the health care system are worth pursuing.

## Introduction

A high proportion of patients with severe mental illness relapse due to nonadherence to psychotropic medication [1-5]. Psychotropic medications, including antipsychotics, antidepressants, and mood stabilizers, are routinely used in the treatment of severe mental illness such as schizophrenia and bipolar disorder [6-8]. Regular attendance at medical services and adherence to the timing, dosage, and frequency of prescribed medication is important in the long-term management of these chronic mental health conditions to avoid the risk of relapse and hospitalization [9]. A recent meta-analysis showed that for patients who discontinued medication after clinical remission, the risk of relapse was 78% at 24 months and 84% at 36 months [10].

In Australia, Community Mental Health Services (CMHS) provide community-based specialized care for people living with severe mental illness as part of a stepped care model [11]. Clinical decision support systems (CDSS) can assist CMHSs in the early detection of nonadherence to facilitate a more proactive model of care to help break cycles of preventable relapse and thus improve health outcomes for people with serious mental illness [12]. In current practice, medication adherence is only routinely monitored for the high-risk medication clozapine [13].

One of the challenges of developing systems for monitoring medication adherence is the siloed nature of health care data in Australia, resulting from the way in which health care funding is managed. Health care funding is split between federal and state governments, resulting in siloed data. Broadly speaking, states fund acute care services, which include CMHS, and the commonwealth funds Medicare universal health insurance coverage, which includes general practitioners (GPs), medication, radiology, and pathology [14]. The development of Australia’s national electronic health record, My Health Record (MyHR), has made it possible to improve data sharing across the health care system. Actionable Intime Insights (AI^2^) is an innovative application using MyHR data for automating service disengagement and nonadherence risk monitoring.

In this paper, our objective is to evaluate the implementation of the AI^2^ application to better understand barriers and enablers to implementing a web-based CDSS to support medication adherence and early intervention in CMHS. To do this, we adopt an approach that combines participatory action research (PAR) with normalization process theory (NPT) [15]. PAR includes the voice of the researchers and captures the collaboration between researchers and clinicians over the course of the pilot implementation [15,16]. NPT has emerged as a framework for understanding complex health care interventions and the implementation of health technologies and electronic health records [17-21]. Underpinning the structure of NPT is the pragmatic need to understand the challenges of introducing CDSS into health care settings [22,23].

## Methods

### Actionable Intime Insights Application

**Figure 1:**
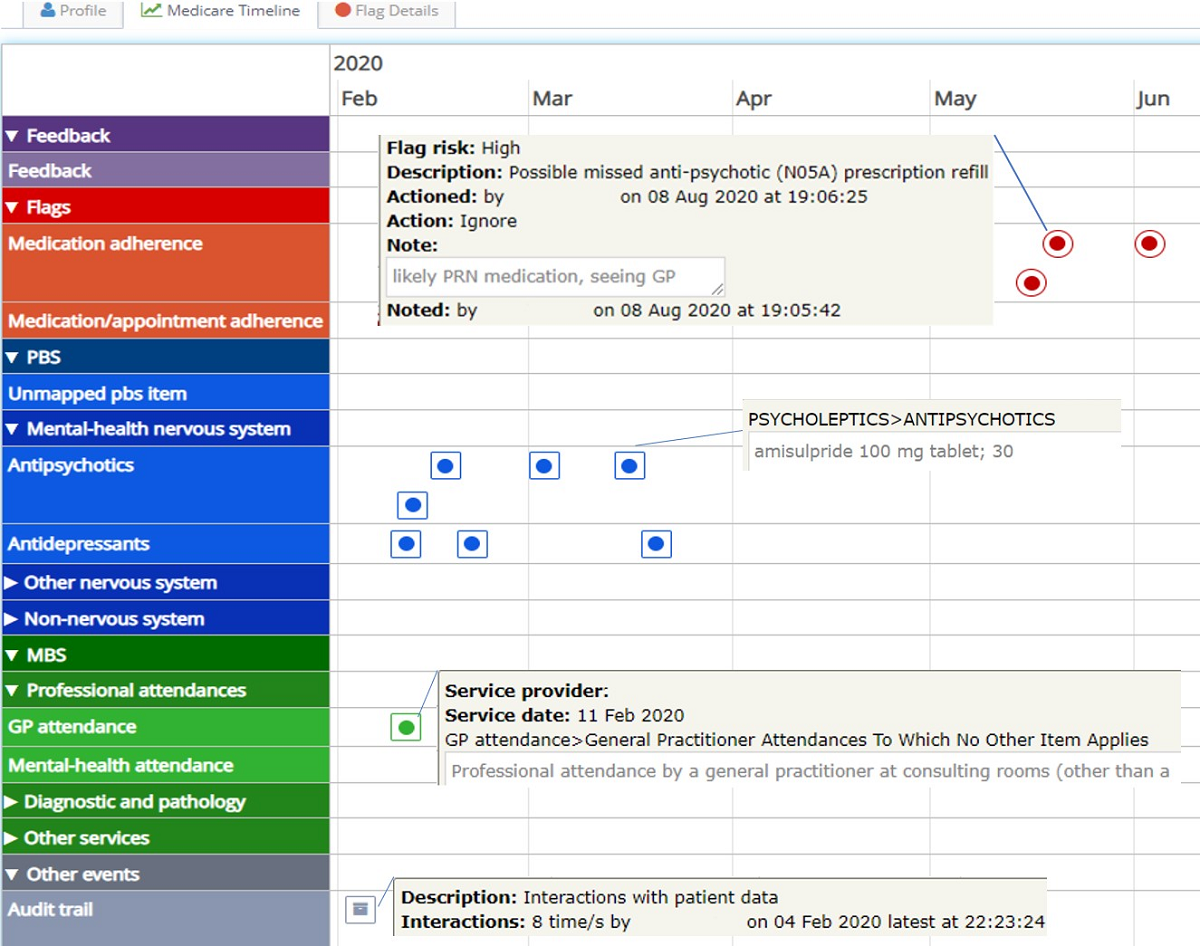
Time line view. GP: general practitioner; MBS: Medicare benefits schedule. PBS: pharmaceuticals benfits schedule; PRN: pro re nata.

Actionable Intime Insights (AI^2^) is a web-based software that synthesizes data from Australia’s national My Health Record (MyHR) system in near real-time to determine whether patients’ records of attendance for medical appointments and prescription refill records reflect treatments appropriate for their condition [24]. Algorithms in the software generate alerts when prescriptions have not been refilled and when 6-month appointments for mental health care plan reviews with the GP have not occurred at the expected times. These alerts are visually presented in a dashboard along with a time line visualization of all previous claims (Figure 1). The decision rules for generating alerts are derived from best practice guidelines for the treatment of schizophrenia, bipolar disorder, and major depression [25,26]. The development of the software, algorithm details, and clinical outcomes of implementation were previously published [24,27,28].

The AI^2^ dashboard lists all clients in the clinic and has a search function to retrieve individual patient data and filters that which can be used to target specific client groups based on episode status (open or closed), risk of nonadherence (high, moderate, or low), and time since last alert. Patient data can be individually reviewed using the time line view (Figure 1).

#### Implementation Site

**Figure 2:**
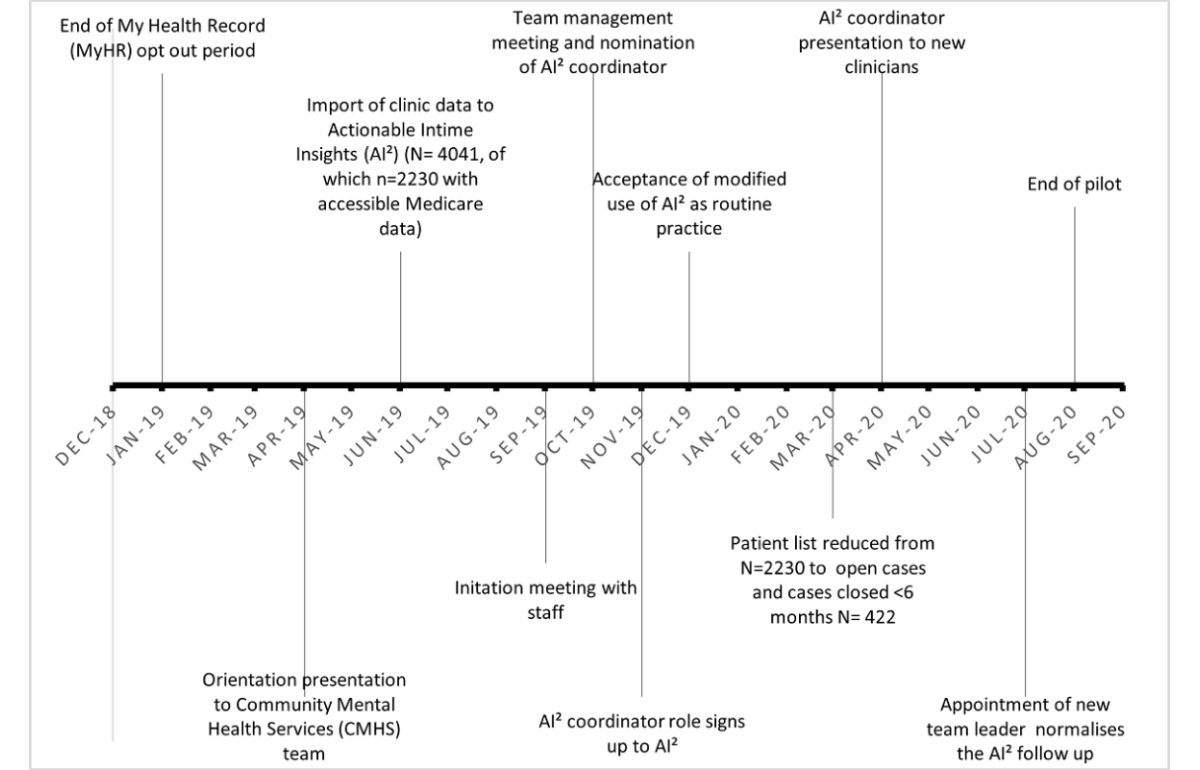
Implementation timeline.

The pilot implementation of AI^2^ was conducted in 2019-2020 in a CMHS in South Australia. The site care team consisted of 9 mental health clinicians from multidisciplinary backgrounds, including 2 on-site psychiatrists (0.4 full-time equivalent [FTE] and 0.2 FTE), 2 nurse consultants, a youth clinician, and a team leader. The service catchment has a highly itinerant population. At the time of implementation, the CMHS was managing 60 case coordination clients, 30 clozapine clients, and around 13 new referrals weekly. Care was provided using a consultation liaison model with psychiatrists consulting with patients on an as-needed basis. Patients were referred for psychiatric care by their GPs or case managers for assessment and advice regarding treatment and services (eg, pharmacotherapy, psychological treatment, anger management, relationship counselling, financial counselling, etc). Following consultation, a written summary of the recommendations including medication advice was sent to the patient and their GP. Case managers provided care coordination for patients within their service, and weekly case review meetings were held with the treating psychiatrist. Patients being treated with clozapine were reviewed every 6 months as per protocol, with all other patients reviewed only as requested (by the case mangers or GPs), typically <1/year. A time line of key milestones is captured in Figure 2.

Patient lists including first name, surname, gender, date of birth, and Medicare number were imported to AI^2^ in June 2019. The AI^2^ software uses the patient identifiers to automatically contact the Healthcare Identifiers Service to obtain the patient’s Individual Healthcare Identifier (IHI). Once a patient has an IHI, the server connects to MyHR and downloads Medicare claims data from MyHR to AI^2^. Data are updated on a weekly basis. A minimum of 2 years of Medicare data are downloaded.

### Participants

Participants (N=18) in the research are shown in Table 1.

**Table 1:** Participants (N=18) in this study.

Participants by type			Values, n
**CMHS^a^ participants**			
	Psychiatrist	1	
	Social worker	4	
	Nurse	2	
	Nurse practitioner	1	
	Admin officer	1	
	Occupational therapist	2	
	Peer review worker	1	
**Research team**			
	Psychiatrists	2	
	Computer scientists	2	
	Implementation researchers	2	

^a^CMHS: Community Mental Health Services.

### Data Collection

Consistent with participatory action research, data collection was a 16-month process from start to end of the pilot. The principal site psychiatrist (0.4 FTE) was also a member of the research team and attended weekly research meetings over the course of the pilot implementation. Data were collected throughout the implementation. Data included in the final analysis are shown in Table 2. Transcripts from team meetings and other work products were used to inform timing and context and provide clarification [29].

**Table 2:** Data used in the analysis by type.

Data type	Number of documents
On-site discussion group	2
Emails	15
One-on-one interviews	3
LHN^a^ emails and docs	3
Research team meetings	1
Total	24

^a^LHN: Local Health Network.

### Ethics Approval

This research was approved by the Southern Adelaide Clinical Human Ethics Research Committee (Ref 177.17) and the Country Health SA governance committee and was granted site approval for the use of AI^2^. Clinicians provided online consent for the use of the software. The research team and participating site clinicians also provided written consent for the audio recording of meetings and interviews as well as for the incorporation of work products related to the use of AI^2^ to be included as data.

### Analysis

Transcripts and documents were uploaded to NVivo software and coded using a deductive thematic analysis approach using the following NPT framework constructs: coherence, cognitive participation, collective action, and reflexive monitoring (Table 3) [30,31]. The problem of overlapping constructs in NPT was challenging [32,33]. Several iterations of coding were required to confirm that the coding was consistent with the NPT framework. Agreement on coding was reached between authors YvK and EL. Team members involved in the implementation reviewed the overall results for accuracy and fidelity.

**Table 3:** Four generative mechanisms of the normalization process theory (adapted from Lloyd et al [34], which is published under Creative Commons Attribution 2.0 International License [35]).

Generative mechanism	Description
Coherence	The sensemaking work that people do individually and collectively when they are faced with the problem of operationalizing some set of practices.
Cognitive participation	The relational work that people do to build and sustain a community of practice around a new technology or complex intervention.
Collective action	The operational work that people do to enact a set of practices, whether these represent a new technology or complex health care interventions.
Reflexive monitoring	The appraisal work that people do to assess and understand the ways that a new set of practices affect them and others around them.

## Results

### Coherence: Sensemaking

Coherence describes the sensemaking work done both individually and collectively by clinicians and administrators, as they worked to integrate AI^2^ into their everyday practice [36]. Sensemaking is a process of understanding and rationalization that impacts subsequent actions and roles [36]. The site psychiatrist worked closely with the researchers on the implementation plan. As part of the implementation process, the site psychiatrist and the research team made 2 presentations to staff, an orientation presentation in April 2019 and a more detailed initiation meeting in September 2019. Author YvK also participated in a team management meeting in October 2019 to discuss and plan the rollout (Figure 2).

#### Investigating Medication Adherence

An important part of the work Community Mental Health clinicians do is understanding patients’ medication and adherence. Clinicians in CMHS use multiple databases to both investigate and manage patients. As one participating clinician (referred to as CL in the quotes) confirmed:

So, when we have a client referred to us, we will often refer to not just our database.

CL9

However, prior to the development of MyHR, CMHS did not have access to Medicare data, which includes pharmacy prescription data as well as Medicare claims data for GP attendance, radiology, and pathology. Clinicians, including both social workers and nurses, routinely make calls to patients GPs and family to investigate medication adherence and GP attendance.

We would normally talk to GPs to try and get a sense of whether people have been compliant…talking to family members, talking to clients themselves and talking to GPs is how we get that information.

CL9

#### Change to Care Model

The detection and flagging of nonadherence using AI^2^ were problematic because it represented a change to the existing care model and specifically a change from a reactive to a more proactive model of care.

We are very, very, very used to only managing clients who are open to us or new referrals…[but] alerting us to someone who might be relapsing, who used to see us but hasn’t for some time, we’re just not used to thinking in that way.

CL9

Nonetheless, clinicians were able to draw parallels between AI^2^ and the systems used for monitoring depot injections and clozapine and were able to recognize the value of early intervention. However, they were concerned about extending monitoring to all patients:

So, we have voluntary clients who might be late for a depot, and we’re alerted to that, and we give them a call and say, “Hey, have you forgotten your depot?”…We see that as a part of relapse prevention...although …[AI^2^] does take it to the next level…monitoring…everybody.

CL10

Nurses were more accepting of AI^2^ than social workers. Nurses are used to working with patients under community treatment orders, clozapine management, and depot injections, whereas social workers have a more supportive role and a different relationship with patients of CMH.

A lot of our clients are voluntary. It is based on trust…if I’m checking the [AI2] data, then making that phone call and saying,“…I’m aware that you haven’t been back to your GP. I don’t feel comfortable around that…I think that changes your relationship with your client.

CL12

### Cognitive Participation: Onboarding

Cognitive participation describes the way in which clinicians engage with AI^2^ and what motivates their use of the software [37,38].

#### Legitimation of Patient Monitoring

Monitoring patients’ adherence was viewed as more acceptable for case-managed (open) patients than for closed patients:

This is all right with clients [who are] case managed…ones [who are] not case managed?…They’ve been discharged back to their primary carer, which is the GP. So, do we still [get] involved with contacting these people saying, “You didn’t go for your script?”

CL1

It also raised concerns about jurisdiction:

Some ethical considerations here also. [For example], I had a red alert for a client that I had transferred to another mental health team some months earlier. I felt obligated to notify the current treating team in the event that client was deteriorating.

CL9

Consistent with the negative media coverage of MyHR leading up to the implementation pilot, several clinicians viewed AI^2^ by association as:

A breach of patient confidentiality, or patient ethics in terms of patients’ rights.

CL9

Clinicians were also concerned about the specific use of AI^2^ for surveillance, with one reference to “Big Brother.” Several clinicians also felt that patients would not expect their MyHR data to be used to detect nonadherence:

It’s a bit of a surprise that that information might be used in that way. Yeah. So, when people opted in to have their records shared, this was one of the things that, maybe, wasn’t on the agenda.

CL3

#### Medicolegal Concerns

While the use of a system based on MyHR data was a concern, clinicians were more troubled by the risk of moral hazard. Other than the site psychiatrist, clinicians refused to sign up to AI^2^ out of concern for the potential medicolegal implications of alerts.

The dilemma really is…a moral hazard…if you find out something, what is your obligation to do with that information?...If I don’t look at them, it’s not [a moral hazard].

CL11

### Collective Action: Enacting

Collective action refers to integrating new practices into existing workflow [34]. It also includes understanding how a new practice impacts interactions between clinicians and patients [39].

#### Phase 1: Getting Started

**Figure 3:**
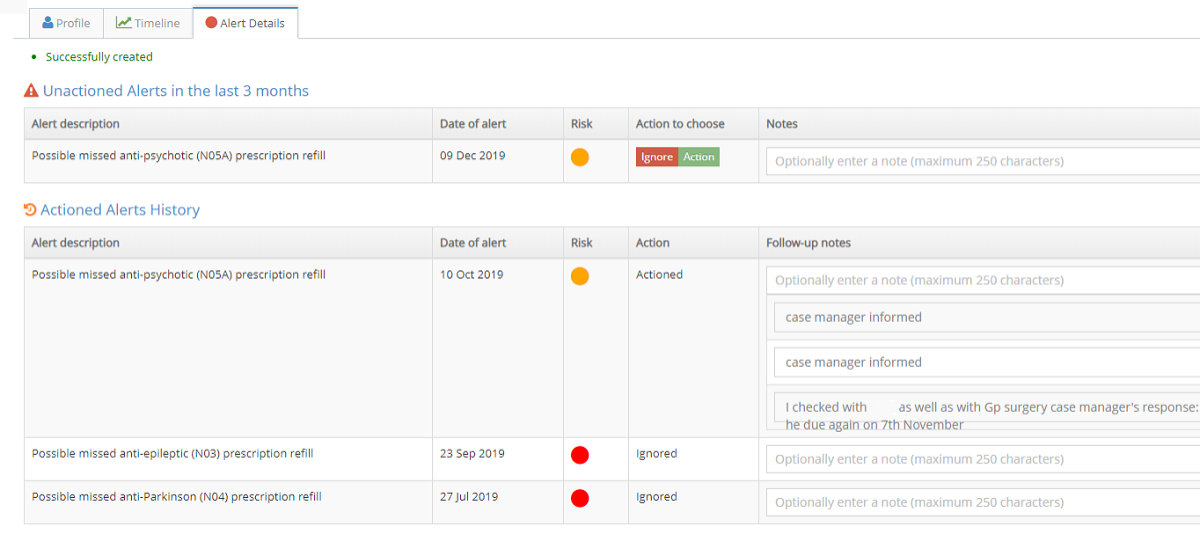
Alerts view.

The original vision for the pilot implementation was that all clinicians would sign up to AI^2^ and use the alerts on the dashboard to provide early intervention, which involved 3 actions: (1) triaging patients with alerts to identify the need for further follow-up; (2) following up on patient alerts identified through the triage process by making phone calls to the patient, the GP, carer, or other listed contacts; and 3) entering follow-up feedback into the software (Figure 3).

Clinicians were initially reluctant to sign up to AI^2^. Therefore, a workaround was devised to get the pilot implementation underway. The psychiatrist agreed to triage the alerts initially. When approaches to the emergency triage and liaison service and a level 4 nurse with a quality and safety portfolio acting at a regional rather than team-based level failed, the psychiatrist continued to triage alerts for the duration of the pilot. The number of alerts generated when patient data were initially uploaded onto AI^2^ was overwhelming. This was because the clinic patient list included both case-managed patients (open) and closed patients. Closed patients were included because they are the primary target for early prevention. Open patients were already receiving care. Another reason for the high number of alerts was that the Medicare data uploaded by AI^2^ contained 2 years of data, creating a backlog of alerts. To make the task more manageable, the psychiatrist made the decision to use dashboard filters to develop a use case focusing on the management of open cases and cases closed within the last 6 months.

The task of following up on alerts was delegated to site-based clinicians, and the psychiatrist encouraged staff and leadership to come up with a workable solution. Resistance to change in practice and the additional work resulting from the introduction of AI^2^ caused ongoing delays in implementation, initially attributable to workload and managing resources.

We were at one point 7.4 FTE down. We’re now sitting at 2.3, 2.4 just over two at the moment…to me it’s…a resource matter. Who is the resource? Where is the resource going to come from within the team? [W]hat impact will that have on the rest of our duties and our tasks?

CL8

#### Phase 2: Role of the AI2 Coordinator

While acknowledging the benefits of AI^2^, the team leader’s priority was to maintain routine work at a time of low staffing levels and competing demands, which resulted in slow progress being made. At a management meeting in October 2019, at which point staffing levels were back to normal, it was decided to appoint an AI^2^ coordinator to assist the psychiatrist and manage patient follow-up. The AI^2^ coordinator was to be a clinician with some availability and flexibility who was able to perform complex work independently and was willing to take AI^2^ on as a portfolio. An additional time allocation of 0.2 FTE was discussed but never implemented due to resourcing priorities set outside the team.

A level 3 nurse at the management meeting agreed to take on the role of AI^2^ coordinator and signed up to AI^2^ in November 2019. With only the psychiatrist signed up to AI^2^, the psychiatrist would log onto AI^2^, triage patients with alerts, and identify patients in need of further follow-up. Since no one else was signed up to AI^2^, the psychiatrist would take a screenshot of the patient’s time line view and email it to the AI^2^ coordinator along with brief notes. The email and time line conveyed the history and the essential information required by clinicians to make follow-up calls with the patients.

**Figure 4:**
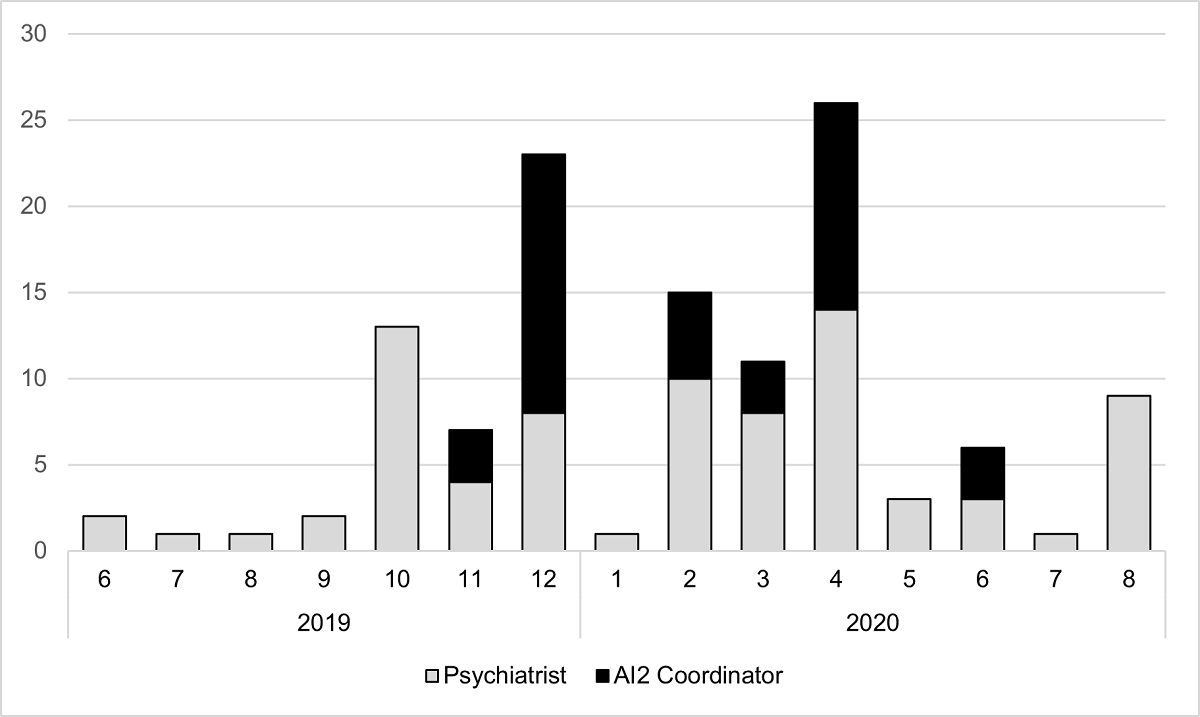
Frequency of use (log in) over the implementation period. AI^2^: Actionable Intime Insights

However, without additional FTE, the AI^2^ coordinator was not always able to follow-up with the patients, so the process was adapted to distribute the follow-up responsibilities to the respective case managers, leaving the coordinator to follow-up on any unassigned patients. The coordinator collated the feedback and entered that into AI^2^. Figure 4 shows the frequency of access to AI^2^ over the course of the pilot. A total of 242 patients were contacted, or attempts to contact them were made.

#### Procedure

Importantly, clinicians agreed on how to document AI^2^ activities in the clinic’s electronic record database. A local work site instruction was developed to document the use of AI^2^ in the clinic (Multimedia Appendix 1). Staff created “referral episodes” for closed patients to documents activities resulting from AI^2^ alerts in the main clinical system. Referral episodes are used in the clinical software to document events such as calls from the police or neighbors.

#### Training Needs

The training mainly focused on how to communicate with patients. Initially, clinicians were concerned about patients’ reactions to cold calls based on alerts received from AI^2^. Making cold calls is part of routine practice when information is received from people in the community or for patients on clozapine, depot, and community treatment orders, but in the wake of negative media coverage of MyHR, clinicians were concerned about patient reactions to calls based on data from MyHR.

Concerns around confidentiality and that this person would be really angry with the call...were the concerns of a number of team members.

CL9

Based on her own experience, the AI^2^ coordinator trained clinicians on making AI^2^ follow-up calls to patients. This training was important for overcoming clinician concerns.

I was… standing with them, looking at the screen shot that [the psychiatrist] had sent…I was saying, if I was to ring this client, I would say—and then I would tell them what I would say.

CL9

#### Normalization

Making follow-up calls became more routine over time.

I don’t think the team believed me until they started making the phone calls themselves and could see that…the client was okay with receiving that call.

CL9

Moreover, despite initial concerns, follow-up calls were well received by patients and viewed as another touchpoint in the trajectory of care, giving patients the feeling of having a safety net.

[Patients] felt reassured that there was an extra layer of protection in a sense of support or monitoring.

CL9

Toward the end of the implementation in July 2020, there was a significant change of attitude in staff and acceptance of AI^2^ as business as usual. Two factors may have contributed to this: (1) growing awareness of the patients’ acceptance of the monitoring and follow-up phone calls and (2) a change from a collaborative leadership style to a more directive leadership.

I think people still hold those views, but the fact that we now have…a team leader who’s quite directive…[and] they’ve now had some exposure and have realized that actually their patients don’t mind the phone call. Those…things lining up together has been what’s, I think, turned things around.

CL9

At the meeting, it was also decided that the task of follow-up on alerts should be spread equally across the team rather than being passed onto the patient’s case manager. This was determined to be a more equitable approach.

### Reflexive Monitoring: Reviewing

Reflexive monitoring refers to the work of appraising change and its impact [17].

#### Time Line View

The reluctance of clinicians to sign up to AI,^2^ especially in the earlier stages of the pilot, prevented clinicians from benefitting from the time line view, which provides a valuable history of patients’ medication and attendance at medical appointments and that is not available on the clinic’s Electronic Health Record (EHR). A routine and often time-consuming part of CMHS clinical work is making calls to patients, carers, GPs, and pharmacists to understand patient medication history and adherence.

Being able to check this on the…dashboard instead [of making calls] will save that time for everyone.

CL9

Having AI^2^ as a separate application to use was not viewed as a problem. Clinicians were accustomed to using multiple databases to research patient history, especially for new referrals.

So, whenever a client is referred to us, it’s helpful to have the information, and people will often refer to those 3 databases to gather collateral.

CL9

The AI^2^ coordinator found that using AI^2^ to investigate adherence was time saving:

We’ve got to ring up the GPs. The GPs don’t always answer because they’re not always there. So, we’re doing a hell of a lot of chasing around and it can—for about three patients, three to four patients—it can take the best part of half a day to do all this, so it’s very time consuming.

CL1

Clinicians also acknowledged that information from GPs is generally limited to what scripts were issued, whereas Medicare data indicates when medications were collected and is a better proximal surrogate indicator of adherence.

Although AI^2^ cannot guarantee compliance with…oral medication, it can certainly give a more accurate representation about what may have been happening.

CL9

Clinicians also discussed the potential of the time line view as a way to:

lead to better conversations with the client about future care planning and how to prevent relapse.

CL9

The value of the time line view was recognized by the Local Health Network who introduced the mandatory use of the time line to inform consumer care planning and service delivery decision making at the 90-day clinical review and the discharge review to supplement information that would otherwise be used in a clinical review process.

#### Missing Data

While all the clinic’s patients are displayed on the dashboard, not all patients’ Medicare data were able to be uploaded. MyHR was designed to give patients control over their MyHR, so patients may have opted out of the MyHR Record or blocked access. There were also systemic issues related to health care funding. Data may not have been uploaded due to invalid or missing Medicare numbers or errors in the date of birth or spelling of the name. CMHS are state funded and do not use Medicare for claims, so patients’ Medicare details can be incomplete. To address this issue, the clinic reception began to routinely ask patients for their Medicare details, a common practice in Medicare based-services such as GP practices.

#### Improving Software

The alerts on AI^2^ are generated by algorithms based on best practice guidelines. Based on clinician feedback on alerts, the pilot testing of these algorithms identified issues that could be improved with further refinement.

#### Follow-up Alerts

Data on AI^2^ is near real-time, meaning that delays in pharmacy uploads to Medicare or Medicare updates to MyHR can appear temporarily as alerts for missing medication; while these alerts are overridden by the system once an upload occurs, they can add to data noise.

Pharmacies, specifically the ones which a lot of our patients get supply from, don't update the PBS record in a timely way; thus their delayed reporting to MyHR is leading to an alert.

CL11

In the alerts that were passed onto the clinicians for follow-up, there were also several false positives and false alerts.

There were some clients who had stopped taking their medication, but the case manager…already knew that that was likely to have been the case. So that wasn’t new information for them, as such…others were false alerts, like, oh yes, the client just told me they have been taking their medication, so I’m not sure why it’s come up on the system.

CL9

Of the alerts followed up on, only a few indicated unsupervised discontinuation of medication. Others indicated that the person was in hospital or in remand, where an alert was generated because medication prescribed in these settings was not funded by Medicare.

[There is] a growing sense among case managers that there is very little return for the effort to chase the alerts, and many people's time is wasted in the process.

CL11

Without a positive case study, the impact of preventative care was difficult for staff to appreciate, but the AI^2^ coordinator remained pragmatic.

Yes, there is another task, but ultimately, it might prevent you from having to do another 10 tasks if this person gets sick and needs detaining and needs to end up in hospital, they would see the value in that. But at the moment, it’s just another sort of task that we’re adding.

CL9

Arguably, the patients reviewed over the course of the trial received improved quality of care, benefitting from a clinical review that would otherwise not have happened. Several patients also received an outreach call from Community Mental Health clinicians.

## Discussion

### Principal Findings

This study evaluates the implementation of an innovative clinical decision support system to support early intervention for Community Mental Health Services using data from Australia’s national electronic record, MyHR. The use of data from MyHR for clinical purposes became a reality in February 2019 at the end of the opt-out period, when MyHRs were automatically created for people who chose not to opt out. At the end of the opt out period, 90.1% of Australians had a MyHR [40].

Medicare data in MyHR are increasingly being used in emergency departments by clinicians and pharmacists because they provide valuable information that is not available to clinicians in hospital databases due to the siloed nature of health care in Australia [41]. AI^2^ is an application designed for CMHS to automate risk monitoring and detection of nonadherence using Medicare data from MyHR to facilitate early intervention and prevent relapse and hospitalization for people living with severe mental illness.

The implementation was a series of ongoing negotiations and compromise resulting in a staggered rollout. At the beginning, we observed significant resistance to restructured work practices as a result of implementing the intervention. The substantial workarounds required to move the implementation forward highlights having the capacity to adapt as an important aspect of implementation. Clinicians’ reluctance to sign up to AI^2^ was attributed to not wanting to be exposed to alerts that potentially create an obligation to act. Clinicians have a duty of care, both ethically and in common law and legislation, whereby they must avoid omissions that could result in harm to others. It could be argued that alerts in AI^2^ are potential early indicators of nonadherence to treatment and medication, which, if not followed up, could result in harm. The pilot implementation allowed researchers to better understand the challenges of implementation and the resources and support needed to implement and sustain a model of care based on early intervention.

### Coherence

The pilot implementation was overshadowed by the negative press coverage leading up to the end of the opt-out period for MyHR, reflecting the impact of broader external influences on implementation [42]. Clinicians struggled with sensemaking in the initial change from explicit to implied consent for clinician access to patient MyHR data [43-45]. Clinicians who routinely make calls to patients based on alerts raised by police or other members of the community expressed real concerns about how patients would react to calls based on alerts from MyHR data. Similarly, clinicians also expressed concerns about the addition of early prevention work in addition to the existing workload, but they were not prepared to use the time line view to save the time involved in ringing around GPs to verify medication adherence, suggesting that the source of the data was likely more challenging than the activities needed to support the implementation. The most significant challenge for clinicians was the concern over the moral hazard associated with the alerts. Exposure to alerts creates a dilemma for clinicians, balancing an opposing ethical/moral duty to follow-up to prevent harm with the management of existing duties, resulting in clinicians refusing to sign up to the software.

Negotiations regarding how to operationalize and proceed with the implementation were protracted, opposing key supporters and detractors. Persistence and persuasion from the psychiatrist were necessary but insufficient to start the implementation. Reluctance by the initial team leader and significant resistance from one team member negatively impacted the attitudes of others, resulting in prolonged delays. Negotiations were resolved with the nominal appointment of the AI^2^ coordinator to work with the psychiatrist and provide direction and leadership from within the Community Mental Health team. While the AI^2^ coordinator was able to monitor and support clinicians to make the follow-up calls, the work was later normalized with a change of team leader.

### Collective Action

Workarounds were needed to operationalize the software. As has been the case in other EHR implementations [46], negative resistance, in the form of clinicians refusing to sign up to AI^2^, resulted in a hindrance workaround [47], whereby the psychiatrist triaged alerts, and the AI^2^ coordinator managed follow-up by allocating the task to others, collected and entered alert feedback on the software and the main clinic database, and provided the necessary training and support. The overwhelming number of alerts generated by the system was also daunting but made more manageable with the development of a specific use case focusing on case-managed (open) and closed patients (<6 months) reflecting the “easy in, easy out” nature of the CMHS.

### Reflexive Monitoring

Like other national EHRs, the aim of MyHR was to support secure patient data sharing across the health care system to improve patient outcomes and reduce the time clinicians spend gathering clinical information [27]. However, despite the potential for change, the uptake and use of MyHR data in acute services has been slow [41,48]. The use of CDSS based on EHRs is growing, and while implementation can be challenging, the potential benefits are improved outcomes for patients through early intervention to prevent relapse and hospitalization and increased efficiency for the health care system. Importantly, for implementation to succeed, a top-down approach is unlikely to work. It is important to have leadership within the team implementing the software to actively support and address issues and concerns of the staff doing the work [33].

### Strengths and Limitations

While this study involved only 1 site, it was important to aid our understanding of the impact of a fundamental change to the Australian health care system and the challenges that lie ahead for the increased use of digital data to enable proactive care. As others have found, pilot implementations are valuable for understanding and ameliorating implementation issues [31]. The strength of the project was in combining use of normalization process theory and participatory action research to drive change, focus on problem solving, and work collaboratively toward achieving an outcome [16]. Working closely with the implementation site and key members of the CMH team also made it possible to gain insight that focus groups or interviews alone would not have afforded.

A weakness of the implementation is the relatively small size of the implementation site, making it potentially more difficult to replicate in larger services. Considerations for future implementations should be to focus on how to manage the very different tasks of triaging and follow-up. Combining triaging and follow-up may result in a duplication of effort in a larger Community Mental Health team. The task of triaging alerts requires a certain level of medical expertise and experience, and while the task is not onerous, it needs to be done routinely to support any given use case. The underlying issues of reluctance to change from proactive to reactive care will always be problematic and is likely best resolved in the medium term with dedicated staff and in the short term with additional resourcing. The underlying concern of moral hazard also needs to be addressed. The easy in, easy out approach to CMHS also means that there is an expectation that closed patients will return, so there is a duty of care that exists between CMHS and their clients, whether they are currently open or closed clients [49]. AI^2^ potentially offers a solution to provide early intervention for closed patients that could reduce relapse and rehospitalization [47].

### Conclusions

The NPT framework has provided a useful structure that clearly identifies the challenges of implementation in a way that can facilitate future improvement. While changes to practice are always challenging, clinicians’ attitudes toward MyHR in the wake of the negative press leading up to the opt-out period impacted the implementation more than we anticipated. Increasing the use of MyHR in emergency services and other areas where siloed data has been an issue will normalize it, allowing clinicians to benefit from having access to data that can make a difference to the lives and well-being of patients. The aim of this pilot implementation was to understand the implementation challenges and test the application design. The pilot was useful in addressing issues with the software and elucidating the challenges of implementing a disruptive software into CMHS [50]. Further trials are needed to determine whether applications like AI^2^ that support early intervention can help reduce overall demand on the already overburdened Community Health Services, but this may require a period of transition that involves additional work for existing staff [51,52].

## Appendix

Multimedia Appendix 1 Local work site instruction.

## Abbreviations

AI^2^: Actionable Intime Insights
CDSS: Clinical Decision Support System
CMHS: Community Mental Health Services
EHR: Electronic Health Record
FTE: full-time equivalent
GP: general practitioner
IHI: Individual Healthcare Identifier
MyHR: My Health Record
NPT: normalization process theory
PAR: participatory action research

## References

[1] Falkai P, Wobrock T, Lieberman J, Glenthoj B, Gattaz WF, Möller Hans-Jürgen, WFSBP Task Force on Treatment Guidelines for Schizophrenia (2005). World Federation of Societies of Biological Psychiatry (WFSBP) guidelines for biological treatment of schizophrenia, Part 1: acute treatment of schizophrenia. World J Biol Psychiatry.

[2] Olivares JM, Sermon J, Hemels M, Schreiner A (2013). Definitions and drivers of relapse in patients with schizophrenia: a systematic literature review. Ann Gen Psychiatry.

[3] Uçok Alp, Polat A, Cakir Sibel, Genç Aysun (2006). One year outcome in first episode schizophrenia. Predictors of relapse. Eur Arch Psychiatry Clin Neurosci.

[4] Ward A, Ishak K, Proskorovsky I, Caro J (2006). Compliance with refilling prescriptions for atypical antipsychotic agents and its association with the risks for hospitalization, suicide, and death in patients with schizophrenia in Quebec and Saskatchewan: a retrospective database study. Clin Ther.

[5] Hasan A, Falkai P, Wobrock T, Lieberman J, Glenthoj B, Gattaz WF, Thibaut F, Möller Hans-Jürgen, World Federation of Societies of Biological Psychiatry (WFSBP) Task Force on Treatment Guidelines for Schizophrenia (2012). World Federation of Societies of Biological Psychiatry (WFSBP) guidelines for biological treatment of schizophrenia, part 1: update 2012 on the acute treatment of schizophrenia and the management of treatment resistance. World J Biol Psychiatry.

[6] Leucht S, Komossa K, Rummel-Kluge C, Corves C, Hunger H, Schmid F, Asenjo Lobos C, Schwarz S, Davis JM (2009). A meta-analysis of head-to-head comparisons of second-generation antipsychotics in the treatment of schizophrenia. Am J Psychiatry.

[7] Sim K, Lau WK, Sim J, Sum MY, Baldessarini RJ (2015). Prevention of relapse and recurrence in adults with major depressive disorder: systematic review and meta-analyses of controlled trials. Int J Neuropsychopharmacol.

[8] Vieta E, Günther O, Locklear J, Ekman M, Miltenburger C, Chatterton ML, Åström M, Paulsson B (2011). Effectiveness of psychotropic medications in the maintenance phase of bipolar disorder: a meta-analysis of randomized controlled trials. Int J Neuropsychopharm.

[9] Kane JM, Kishimoto T, Correll CU (2013). Non-adherence to medication in patients with psychotic disorders: epidemiology, contributing factors and management strategies. World Psychiatry.

[10] Fusar-Poli P, Cappucciati M, Bonoldi I, Hui LMC, Rutigliano G, Stahl DR, Borgwardt S, Politi P, Mishara AL, Lawrie SM, Carpenter WT, McGuire PK (2016). Prognosis of brief psychotic episodes: a meta-analysis. JAMA Psychiatry.

[11] Perkins D (2016). Stepped care, system architecture and mental health services in Australia. Int J Integr Care.

[12] Dea RA (2000). The integration of primary care and behavioral healthcare in northern California Kaiser-Permanente. Psychiatr Q.

[13] Kar N, Barreto S, Chandavarkar R (2016). Clozapine monitoring in clinical practice: beyond the mandatory requirement. Clin Psychopharmacol Neurosci.

[14] Lupton D (2017). Digital health now and in the future: findings from a participatory design stakeholder workshop. Digit Health.

[15] Rudin RS, Bates DW (2014). Let the left hand know what the right is doing: a vision for care coordination and electronic health records. J Am Med Inform Assoc.

[16] Leykum LK, Pugh JA, Lanham HJ, Harmon J, McDaniel RR (2009). Implementation research design: integrating participatory action research into randomized controlled trials. Implement Sci.

[17] May C, Finch T, Mair F, Ballini L, Dowrick C, Eccles M, Gask L, MacFarlane A, Murray E, Rapley T, Rogers A, Treweek S, Wallace P, Anderson G, Burns J, Heaven B (2007). Understanding the implementation of complex interventions in health care: the normalization process model. BMC Health Serv Res.

[18] Scantlebury A, Sheard L, Watt I, Cairns P, Wright J, Adamson J (2017). Exploring the implementation of an electronic record into a maternity unit: a qualitative study using normalisation process theory. BMC Med Inform Decis Mak.

[19] Elwyn G, Légaré France, van der Weijden Trudy, Edwards A, May C (2008). Arduous implementation: does the normalisation process model explain why it's so difficult to embed decision support technologies for patients in routine clinical practice. Implement Sci.

[20] Pope C, Halford S, Turnbull J, Prichard J, Calestani M, May C (2013). Using computer decision support systems in NHS emergency and urgent care: ethnographic study using normalisation process theory. BMC Health Serv Res.

[21] McCrorie C, Benn J, Johnson OA, Scantlebury A (2019). Staff expectations for the implementation of an electronic health record system: a qualitative study using normalisation process theory. BMC Med Inform Decis Mak.

[22] May C, Finch T (2009). Implementing, embedding, and integrating practices: an outline of normalization process theory. Sociology.

[23] Mair FS, May C, O’Donnell C, Finch T, Sullivan F, Murray E (2012). Factors that promote or inhibit the implementation of e-health systems: an explanatory systematic review. Bull. World Health Organ.

[24] Knight A, Jarrad GA, Schrader GD, Strobel J, Horton D, Bidargaddi N (2018). Monte Carlo simulations demonstrate algorithmic interventions over time reduce hospitalisation in patients with schizophrenia and bipolar disorder. Biomed Inform Insights.

[25] Galletly C, Castle D, Dark F, Humberstone V, Jablensky A, Killackey E, Kulkarni J, McGorry P, Nielssen O, Tran N (2016). Royal Australian and New Zealand College of Psychiatrists clinical practice guidelines for the management of schizophrenia and related disorders. Aust N Z J Psychiatry.

[26] Malhi GS, Bassett D, Boyce P, Bryant R, Fitzgerald PB, Fritz K, Hopwood M, Lyndon B, Mulder R, Murray G, Porter R, Singh AB (2015). Royal Australian and New Zealand College of Psychiatrists clinical practice guidelines for mood disorders. Aust N Z J Psychiatry.

[27] Bidargaddi N, van Kasteren Y, Musiat P, Kidd M (2018). Developing a third-party analytics application using Australia's National Personal Health Records System: case study. JMIR Med Inform.

[28] Bidargaddi N, Schrader G, Myles H, Schubert KO, van Kasteren Y, Zhang T, Bastiampillai T, Roughead E, Strobel J (2021). Demonstration of automated non-adherence and service disengagement risk monitoring with active follow-up for severe mental illness. Aust N Z J Psychiatry.

[29] Higgins A, Murphy R, Downes C, Barry J, Monahan M, Hevey D, Kroll T, Doyle L, Gibbons P (2020). Factors impacting the implementation of a psychoeducation intervention within the mental health system: a multisite study using the consolidation framework for implementation research. BMC Health Serv Res.

[30] Elo S, Kyngäs H (2008). The qualitative content analysis process. J Adv Nurs.

[31] Murray E, Treweek S, Pope C, MacFarlane A, Ballini L, Dowrick C, Finch T, Kennedy A, Mair F, O'Donnell C, Ong BN, Rapley T, Rogers A, May C (2010). Normalisation process theory: a framework for developing, evaluating and implementing complex interventions. BMC Med.

[32] McEvoy R, Ballini L, Maltoni S, O'Donnell Catherine A, Mair FS, Macfarlane Anne (2014). A qualitative systematic review of studies using the normalization process theory to research implementation processes. Implement Sci.

[33] Leon N, Lewin S, Mathews C (2013). Implementing a provider-initiated testing and counselling (PITC) intervention in Cape town, South Africa: a process evaluation using the normalisation process model. Implement Sci.

[34] Lloyd A, Joseph-Williams N, Edwards A, Rix A, Elwyn G (2013). Patchy 'coherence': using normalization process theory to evaluate a multi-faceted shared decision making implementation program (MAGIC). Implement Sci.

[35] Attribution 2.0 International (CC BY 2.0). Creative Commons.

[36] Weick KE, Sutcliffe KM, Obstfeld D (2005). Organizing and the Process of Sensemaking. Organization Science.

[37] Patel B, Usherwood T, Harris M, Patel A, Panaretto K, Zwar N, Peiris D (2018). What drives adoption of a computerised, multifaceted quality improvement intervention for cardiovascular disease management in primary healthcare settings? A mixed methods analysis using normalisation process theory. Implement Sci.

[38] Finch TL, Mair FS, O'Donnell Catherine, Murray E, May CR (2012). From theory to 'measurement' in complex interventions: methodological lessons from the development of an e-health normalisation instrument. BMC Med Res Methodol.

[39] Murray E, Burns J, May C, Finch T, O'Donnell C, Wallace P, Mair F (2011). Why is it difficult to implement e-health initiatives? A qualitative study. Implement Sci.

[40] 9 out of 10 Australians have a My Health Record. Australian Digital Health Agency.

[41] Mullins AK, Morris H, Enticott J, Ben-Meir M, Rankin D, Mantripragada K, Skouteris H (2021). Use of My Health Record by clinicians in the emergency department: an analysis of log data. Front Digit Health.

[42] Liberati EG, Ruggiero F, Galuppo L, Gorli M, González-Lorenzo Marien, Maraldi M, Ruggieri P, Polo Friz H, Scaratti G, Kwag KH, Vespignani R, Moja L (2017). What hinders the uptake of computerized decision support systems in hospitals? A qualitative study and framework for implementation. Implement Sci.

[43] Mendelson D, Compagnucci Corrales, Forgó M, Kono N, Teramoto S, Vermeulen E.P.M. (2020). Legal tech and the new sharing economy. Perspectives in Law, Business and Innovation.

[44] Pang PC, McKay D, Chang S, Chen Q, Zhang X, Cui L (2020). Privacy concerns of the Australian My Health Record: implications for other large-scale opt-out personal health records. Inf Process Manag.

[45] Van Kasteren Y, Maeder A, Williams P, Damarell R (2017). Consumer perspectives on My Health Record: a review. Stud Health Technol Inform.

[46] Cresswell KM, Worth A, Sheikh A (2012). Integration of a nationally procured electronic health record system into user work practices. BMC Med Inform Decis Mak.

[47] Ferneley EH, Sobreperez P (2017). Resist, comply or workaround? An examination of different facets of user engagement with information systems. EJIS.

[48] Kosari S, Yee KC, Mulhall S, Thomas J, Jackson SL, Peterson GM, Rudgley A, Walker I, Naunton M (2020). Pharmacists' perspectives on the use of My Health Record. Pharmacy (Basel).

[49] Australian And New Zealand College Of Psychiatrists Clinical Practice Guidelines Team For The Treatment Of Schizophrenia And Related Disorders R (2005). Royal Australian and New Zealand College of Psychiatrists clinical practice guidelines for the treatment of schizophrenia and related disorders. Aust N Z J Psychiatry.

[50] Hwang J, Christensen CM (2008). Disruptive innovation in health care delivery: a framework for business-model innovation. Health Aff (Millwood).

[51] Maguire PA, Looi JC (2022). Moral injury and psychiatrists in public community mental health services. Australas Psychiatry.

[52] RANZCAP (2022). Mental health experts welcome SA Labor’s $182m health investment pledge. RANZCP.

