# Supplementary material for: Automated Decision Support For Community Mental Health Services Using National Electronic Health Records: Qualitative Implementation Case Study
Source: JMIR Hum Factors. 2022 Jul 5;9(3):e35403. doi: 10.2196/35403 (PMC9297136; doi:10.2196/35403)
Supplement: Multimedia Appendix 1 [file humanfactors_v9i3e35403_app1.docx]

# Appendix A

## AI squared integration into the workflow in CMHTs

Local work site instruction

**Introduction:**

The aim of this research project is to provide timely and individualised care for people before relapse or hospitalisation.

AI^2^ is a software platform designed to address the problem of deteriorating mental health/ relapses when people stop taking their medication, or when their medication is not being adequately supervised.

The AI^2^ system notifies case managers through alerts so they can contact and advise their patients about any problems with their care.

**Procedure:**

| **What** | **How** | **Comment clinical** | **Comments research** |
| --- | --- | --- | --- |
| A **senior staff member** (CNC or dedicated project clinician) **checks alerts weekly** | Alerts are based on clinical judgement and historical individual patterns **ignored** or **actioned** |  | An implementation where every staff member accesses the dashboard was abandoned due to staff ambiguities, thus resulting in a single point model (with redundancy provision) |
| **Responding** to alerts | ignore | If the alert appears to be the result of the sensitivity setting in someone who has a somewhat irregular pattern picking up medication but averaged out seems compliant | The signal/noise ratio will need to be refined by adaptations to the algorithm |
|  | action | Based on risk assessment the assertiveness of follow up may vary. |  |
| **Actioning** alerts | AI^2^ alerts are made an agenda item on the morning team meeting. CNC presents alerts as data point of concern that will be triaged further by respective case manager | Case manager follows up:  Talking to patient, carer and/or GP.  The conversation needs to be done sensitively with a care motive evident rather than a control motive as patients are sensitive to perceived paternalism.  Action is recorded as per usual in clinical notes (CCC), however the action also needs to be recorded AI^2^ |  |
| **Closing the loop** between clinical notes and AI^2^ | Case manager who actioned the alert is sending CNC (point person for research) a copy of the entry which is then entered by CNC into the AI^2^ shared dashboard |  | Search functions on AI^2^ website need developing as currently interventions are captured on an excel sheet which is procedurally unsatisfactory. |
| **Reporting a KPI** | Keeping record of actions initiated and actions closed with clinical comment regarding potential relapse avoided |  | Capturing perceived narrative of being helpful or intrusive from a patient perspective. Training requirements to refine the message/conversation |
